# Dataset to explore factors affecting COVID-19 vaccination intention. Evidence from Morocco

**DOI:** 10.1016/j.dib.2022.108365

**Published:** 2022-06-09

**Authors:** Mohammed Abdessadek, Hayat Ben-Saghroune, Omar Boubker, Imane Iken, Houssni Abid, Belkassem El Amraoui, Youssef Khabbal

**Affiliations:** a Laayoune Higher School of Technology, Ibn Zohr University, Morocco; b Laboratory of anesthesia-intensive care and emergency medicine Medical Center of Biomedical and Translational Research Physiology, Pharmacology and Health, Faculty of Medicine and Pharmacy of Fez; c Poison control and pharmacovigilance, Center of Morocco, Rabat, Morocco; d Higher Institute of Nursing Professions and Health Techniques Health Sciences, Laayoune; e Polydisciplinary Faculty of Taroudant, Ibn Zohr University, Taroudant, Morocco; f Faculty of Medicine and Pharmacy, Ibn Zohr University, Agadir, Morocco

**Keywords:** Coronavirus, Psychological antecedents, Health belief model, Vaccination intention

## Abstract

The coronavirus pandemic (COVID-19) has had an immense impact on humanity in every aspect of life. Governments around the world have mandated movement restrictions, including in the Moroccan government, in which unfortunately the pandemic continues to propagate and causes serious problems for public health and economic activities in the Kingdom. As a major factor in the fight against the spread of COVID-19, the Moroccan government has undertaken major efforts to ensure the availability of the COVID-19 vaccines for all citizens. These valuable efforts resulted in initiation of the vaccination campaign, which started on February 14, 2021. As vaccination was voluntary, identifying the factors promoting citizen's intention to take the vaccine against COVID-19 may help government to take additional precautions to address the propagation of COVID-19, and ensure a return to normal life. Hence, this data article aims to identify factors influencing the Moroccan citizens to get a vaccine for COVID-19. The data were collected using an online questionnaire among Moroccan citizens. In addition, the Partial Least Squares Structural Equation Modeling technique was adopted in order to analyze the collected dataset.

## Specifications Table

**Table:** 

Subject	Biological sciences
Specific subject area	Immunology
Type of data	Table, Image, Graph, and Figure
How data were acquired	An online questionnaire was carried out among Moroccan citizens.
Data format	Raw, analyzed, filtered, and descriptive data
Parameters for data collection	The survey was self-administered using Google Forms tools during the months of Mai and October 2021.
Description of data collection	The questionnaire link was shared using social networks, including Telegram, WhatsApp, and Facebook.
Data source location	Morocco; Latitude: 31.7945; Longitude: ‒7.0849.
Data accessibility	Repository name: Mendeley Data Data identification number (doi): 10.17632/vgggrsmj89.1 Direct URL to data: https://data.mendeley.com/datasets/vgggrsmj89/1

## Value of the Data

• The dataset can be used to identify factors affecting COVID-19 vaccination intention.
• This dataset provides insights into diverse aspects of the health belief model and theory of planned behaviour.
• This dataset can be employed to enlighten public authorities (Moroccan Ministry of Health) on the importance of the health belief model variables and psychological antecedents of vaccination as key factors to enhance attitude toward vaccination and COVID-19 vaccination intention.
• This dataset can serve as a valuable guideline for identifying factors that drive intent to vaccinate against COVID-19 in developing countries with similar socio-cultural and economic factors to Morocco.

## 1 Data Description

The measuring scales included in the current data article were selected from prior studies. Accordingly, the constructs of health belief model constructs [1], were gauged through four sub-components, namely perceived susceptibility (2-items), perceived severity (2-items), perceived benefits (3-items), and perceived barriers (5-items). Likewise, the 5 C psychological antecedents of vaccination were scored using 14 items [1], including confidence (3-items), constraints (3-items), complacency (3-items), calculation (3-items), and collective responsibility (2 items). Regarding operationalization of the latent constructs of theory of planned behaviour [1,2], we selected 3 items for measuring attitude toward COVID-19 vaccine, 2 items to measure subjective norm (2-items), and a single item to operationalize both behavioral control and vaccination intention (Table 1). The selection of these measurement scales is motivated by their empirical reliability, as they have been frequently applied in a variety of research contexts. Survey participants were instructed to rate items according to a 5-point Likert scale, which ranged from strongly disagree to strongly agree.

**Table 1:** Operationalization of the constructs.

	Variables	Items	Code
Health Belief Model	Perceived susceptibility	I am worried about the likelihood of getting infected by COVID-19.	HBM-Sus1
		I am at high risk of COVID-19 because of my health conditions.	HBM-Sus2
	Perceived severity	I will be very sick if I get infected by COVID-19.	HBM-Sev1
		I am very concerned that I could die from COVID-19.	HBM-Sev2
	Perceived benefits	I think vaccination is good because it will make me less worried about COVID-19.	HBM-Ben1
		I believe vaccination will decrease my risk of getting infected by COVID-19.	HBM-Ben2
		I think the complications of COVID-19 will decrease if I get vaccinated and then get infected with Coronavirus.	HBM-Ben3
	Perceived barriers	I am worried that the possible side effects of the COVID-19 vaccination would interfere with my usual activities.	HBM-Bar1
		I am concerned about the efficacy of the COVID-19 vaccine.	HBM-Bar2
		I have a concern that I may receive faulty/fake COVID-19 vaccine.	HBM-Bar3
		It concerns me that the development of a COVID-19 vaccine is too rushed to test its safety properly.	HBM-Bar4
		I am concerned about the long-term side effects of the COVID-19 vaccination.	HBM-Bar5

5C psychological antecedents of vaccination	Confidence	I am completely confident that COVID-19 vaccines are safe.	PAV-Conf1
		I am completely confident that COVID-19 vaccines are effective.	PAV-Conf2
		Regarding COVID-19 vaccines, I am confident that public authorities decide in the best interest of the community.	PAV-Conf3
	Constraints	Everyday work stress may prevent me from getting vaccinated.	PAV-Cons1
		For me, it is inconvenient to receive vaccinations.	PAV-Cons2
		Visiting the doctors makes me feel uncomfortable; this keeps me from getting vaccinated.	PAV-Cons3
	Complacency	I think it is unnecessary to receive vaccinations, as it cannot prevent COVID-19.	PAV-Com1
		I believe my immune system is powerful; it will protect me from COVID-19.	PAV-Com2
		I believe COVID-19 is not much a severe disease that I should get vaccinated against it.	PAV-Com3
	Calculation	When I think about getting vaccinated against COVID-19, I weigh the benefits and risks to make the best decision possible.	PAV-Cal1
		When I think about getting vaccinated against COVID-19, I will first consider whether it is effective or not.	PAV-Cal2
		Before I get COVID-19 vaccinated, I need to know about this vaccine in detail.	PAV-Cal3
	Collective responsibility	I will take COVID-19 vaccine because, in that way, I can protect people with a weaker immune system.	PAV-Res1
		I think vaccination against COVID-19 is a collective action to prevent the spread of diseases.	PAV-Res2

Theory of Planned Behaviour	Attitude toward COVID-19 vaccine	I think the COVID-19 vaccination is necessary.	ATCov1
		I think the COVID-19 vaccination is a good idea.	ATCov2
		I think the COVID-19 vaccination is beneficial.	ATCov3
	Subjective norm	My family members will support me to get vaccinated against COVID-19.	SN1
		People whose opinion I care about would say that it is a good idea for me to get vaccinated against COVID-19.	SN2
	Behavioral control	If I want, I can register for COVID-19 vaccination.	PBC1
	Vaccination intention	How likely are you to vaccinate against COVID-19?	Int1

As indicated in Table 2, the study sample comprised more females (N= 175; 54.2%) than males (N= 148; 45.8%), and most of them were between 20 and 39 years old (84.9%). Furthermore, the majority of responses (61.7%) were from the cities of Laayoune (23.3%), Fez (17.6%), Taounate (8.7%), Agadir (6.5%) and Casablanca (5.6%). 140 of the respondents were government employees (43.3%), and 123 of them were students (38.1%). 53.3% of participants were graduate students while 41.5% were Masters or PhD level. Additionally, a large part of the survey participants has had a monthly income ranging from 5000 to 15000 Moroccan dirhams (43.4%). Concerning vaccine preference, a large proportion of respondents have been vaccinated with Sinopharm COVID-19 vaccine (57.0%) followed by AstraZeneca COVID-19 vaccine (20.7%).

**Table 2:** Respondents characteristics (N = 323).

Profile	Characteristic	Frequency	Percentage
Gender	Male	148	45.8%
	Female	175	54.2%

Age	20 to 29	172	53.3%
	30 to 39	102	31.6%
	40 to 49	25	7.7%
	50 to 59	18	5.6%
	60 and older	6	1.9%

Marital status	Unmarried	137	42.4%
	Married	128	39.6%
	Widowed or divorced	58	18.0%

Education level	Secondary and higher secondary	17	5.3%
	Graduate (BAC+2 or BAC+3)	172	53.3%
	Masters or PhD	134	41.5%

Occupation	Government employee	140	43.3%
	Student	123	38.1%
	Private sector employee	31	9.6%
	No occupation	16	5.0%
	Business owners	7	2.2%
	Retired	6	1.9%

Monthly income	No response	128	39.6%
	Less than 5 000	34	10.5%
	5000 -10 000	51	15.8%
	10 000 - 15 000	89	27.6%
	More than 15 000	21	6.5%

Vaccine preference	No response	60	18.6%
	Sinopharm	184	57.0%
	AstraZeneca	67	20.7%
	Pfizer	5	1.5%
	Johnson & Johnson	4	1.2%
	Sinovac	2	0.6%
	Moderna	1	0.3%

Given that science provides a valuable benefit to the economy of developing countries [3], the purpose of this dataset is to identify motivating factors for citizens to be vaccinated against COVID-19, which will help to guide policymakers' future efforts to proactively develop vaccination programs for increasing vaccination intention among Moroccans citizens. Experts call for an interdisciplinary strategy for absorbing and filtering information to help understand factors influencing vaccine hesitancy [4]. At this level, this dataset aims to identify factors that may reduce vaccine hesitancy, by considering the theory of planned behaviour (TPB), the health belief model (HBM), and the 5c psychological antecedents of vaccination.

## 2 Experimental Design, Materials and Methods

**Fig. 1:**
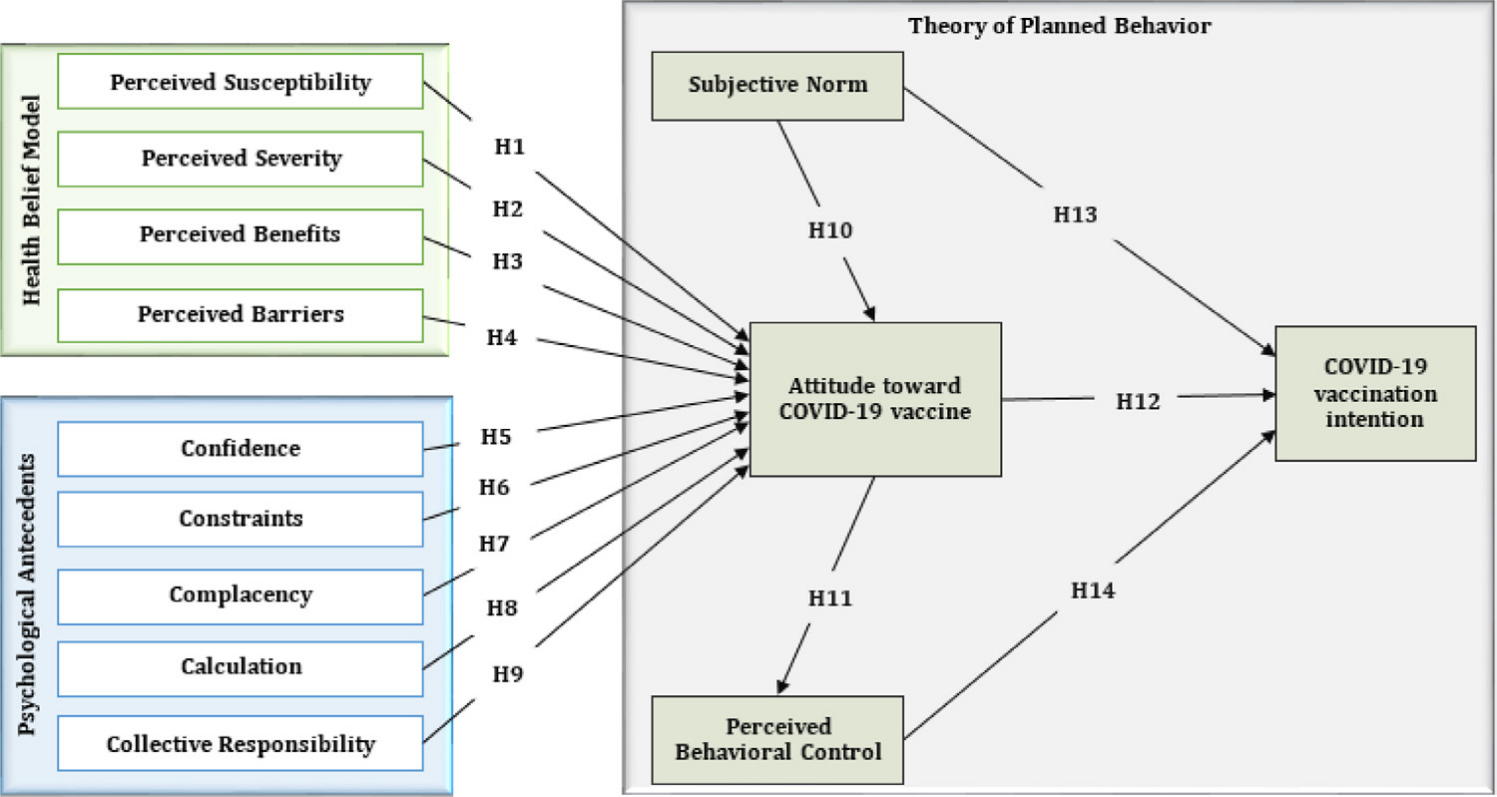
Conceptual model.

Our research model was developed using the HBM, the 5 C psychological antecedents of vaccination and the TPB. As indicated in Fig. 1, the health belief model including perceived susceptibility (H1), perceived severity (H2), perceived benefits (H3), and perceived barriers (H4) directly affects individual attitudes toward COVID-19 vaccination. In addition, the 5 C psychological antecedents of vaccination, i.e., confidence (H5), constraints (H6), complacency (H7), calculation (H8), and collective responsibility (H9) directly and significantly influence on attitudes toward COVID-19 vaccination. Likewise, the conceptual model assumes that individual attitude toward vaccination depends on subjective norm (H10). Further, attitude toward vaccination positively influences on perceived behavioural control (H11). These three variables, i.e., attitude (H12), subjective norm (H13), and perceived behavioural control (H14) are assumed to have a direct and positive impact on people's intention to be immunized against COVID-19.

The survey is designed around two parts. The first part is focused on gathering data on citizens' socio-demographic profiles, including gender, age, marital status, education level, occupation, city, monthly income, and vaccine preference. The second part of the questionnaire is designed to capture data related to latent variables including perceived susceptibility, perceived severity, perceived benefits, perceived barriers, confidence, constraints, complacency, calculation, collective responsibility, attitude toward COVID-19 vaccine, subjective norms, behavioral control, and vaccination intention.

To improve the comprehensibility of the survey questions, the developed questionnaire was pre-tested among ten government employees. The original questionnaire (English version) has been translated into Arabic and French by language specialists. Hence, respondents were allowed to select one of the three languages i.e., English, Arabic, or French to fill in the questionnaire.

The questionnaire has been administered through Google Forms, as well as the questionnaire link has been shared using social networks, including Telegram, Facebook, and WhatsApp. Hence, the survey was conducted through an online process among Moroccan citizens over a period of six months (From May to October 2021) and a total of, 323 valid responses have been obtained.

The gathered dataset was handled through Microsoft Excel in order to generate descriptive statistics on socio-demographic profiles of survey participants. While the collected data for the different latent variables were stored and coded in a comma-separated values (CSV) file which is compatible with the SmartPLS software.

To verify the research model, we first operationalized the latent constructs (1), then elaborated and pretested the research questionnaire (2), followed by assembling data (3), and finally analyzing the collected dataset based on the structural equation modeling based on a partial least squares (PLS-SEM) approach using the SmartPLS program. At this level, the PLS-SEM approach was employed in order to verify the hypotheses.

**Fig. 2:**
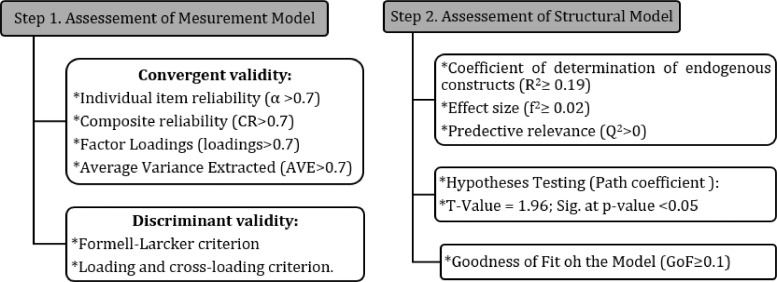
Data analysis steps using PLS-SEM.

As indicated in Fig. 2, this approach required two complementary steps: the evaluation of the measurement models, and the validation of the structural model [5]. More accurately, the measurement models' assessment involves the checking of convergent validity across loading, Cronbach's alpha, composite reliability, and average variance extracted, whereas the checking of discriminant validity is based on the Fornell-Larcker and the Heterotrait-Monotrait Ratio (HTMT) criteria. Moreover, evaluating the inner model entails verifying the coefficient of determination, the effect size, the predictive relevance, and model goodness-of-fit [6].

As shown in Table 3, all items loading values are significantly higher than 0.71, which meet the scientific standards [7]. In addition, the values of Cronbach's alpha (α), reliability (ρA), composite reliability (ρc), and average variance extracted (AVE) are respectively higher than 0.74, 0.84, 0.85, and 0.65.

**Table 3:** Convergent validity.

Variables		Code	Loading	α	rho_A	CR	AVE
Health Belief Model	Perceived Susceptibility	HBM-Sus1	0.98	0.96	0.96	0.98	0.96
		HBM-Sus2	0.98				
	Perceived Severity	HBM-Sev1	0.94	0.75	0.86	0.89	0.79
		HBM-Sev2	0.84				
	Perceived Benefits	HBM-Ben1	0.92	0.87	0.89	0.92	0.80
		HBM-Ben2	0.85				
		HBM-Ben3	0.91				
	Perceived Barriers	HBM-Bar1	0.75	0.87	0.89	0.90	0.66
		HBM-Bar2	0.84				
		HBM-Bar3	0.81				
		HBM-Bar4	0.83				
		HBM-Bar5	0.81				

5C psychological antecedents of vaccination	Confidence	PAV-Conf1	0.92	0.86	0.86	0.92	0.78
		PAV-Conf2	0.92				
		PAV-Conf3	0.82				
	Constraints	PAV-Cons1	0.71	0.74	0.84	0.85	0.65
		PAV-Cons2	0.90				
		PAV-Cons3	0.80				
	Complacency	PAV-Com1	0.89	0.79	0.92	0.87	0.69
		PAV-Com2	0.75				
		PAV-Com3	0.84				
	Calculation	PAV-Cal1	0.86	0.84	0.88	0.90	0.76
		PAV-Cal2	0.85				
		PAV-Cal3	0.90				
	Collective Responsibility	PAV-Res1	0.95	0.90	0.91	0.95	0.91
		PAV-Res2	0.96				

Theory of Planned Behaviour	Attitude toward COVID-19 vaccine	ATCov1	0.95	0.95	0.95	0.97	0.91
		ATCov2	0.96				
		ATCov3	0.96				
	Subjective Norm	SN1	0.94	0.86	0.87	0.94	0.88
		SN2	0.94				
	Behavioral Control	PBC1	1.00	1.00	1.00	1.00	1.00
	Vaccination intention	Int1	1.00	1.00	1.00	1.00	1.00

Table 4 demonstrates the discriminant validity of outer models based on the Fornell-Larcker criterion, showing that the AVE of each latent construct is higher than the construct's highest squared correlation with any other latent construct [8].

**Table 4:** Discriminant validity based on Fornell-Larcker criterion.

	ATCov	HBM-Bar	HBM-Ben	Int	PAV-Cal	PAV-Res	PAV-Com	PAV-Conf	PAV-Cons	PBC	HBM-Sev	SN	HBM-Sus
Attitude toward COVID-19 vaccine	**0.95**												
Barriers	‒0.20	**0.81**											
Benefits	0.69	‒0.15	**0.89**										
COVID-19 vaccination intention	0.56	0.03	0.47	**1.00**									
Calculation	0.17	0.38	0.06	0.21	**0.87**								
Collective Responsibility	0.75	‒0.16	0.68	0.52	0.18	**0.95**							
Complacency	‒0.45	0.37	‒0.36	‒0.19	0.25	‒0.38	**0.83**						
Confidence	0.69	‒0.34	0.61	0.45	0.06	0.61	‒0.28	**0.88**					
Constraints	‒0.40	0.34	‒0.35	‒0.20	0.16	‒0.37	0.67	‒0.24	**0.81**				
PBC	0.42	0.01	0.33	0.82	0.19	0.36	‒0.16	0.33	‒0.13	**1.00**			
Severity	0.15	0.20	0.10	0.10	0.17	0.12	‒0.04	0.08	0.07	0.10	**0.89**		
Subjective Norm	0.60	‒0.01	0.50	0.58	0.21	0.58	‒0.20	0.53	‒0.21	0.41	0.10	**0.94**	
Susceptibility	0.13	0.30	0.07	0.11	0.18	0.10	‒0.03	0.03	0.07	0.03	0.45	0.17	**0.98**

As an alternative to Fornell-Larcker criterion, the HTMT offers a better assessment criterion to verify outer models' discriminant validity. Accordingly, the higher HTMT value was 0.82 (Table 5), which meet the specialist recommendations [9].

**Table 5:** Discriminant validity of outer models using the HTMT criterion.

	ATCov	HBM-Bar	HBM-Ben	Int	PAV-Cal	PAV-Res	PAV-Com	PAV-Conf	PAV-Cons	PBC	HBM-Sev	SN	HBM-Sus
Attitude toward COVID-19 vaccine													
Barriers	0.21												
Benefits	0.76	0.16											
COVID-19 vaccination intention	0.57	0.11	0.50										
Calculation	0.18	0.45	0.08	0.21									
Collective Responsibility	0.81	0.17	0.77	0.55	0.20								
Complacency	0.47	0.39	0.37	0.18	0.30	0.39							
Confidence	0.77	0.39	0.71	0.48	0.07	0.69	0.27						
Constraints	0.45	0.38	0.39	0.22	0.19	0.43	**0.82**	0.27					
PBC	0.43	0.09	0.35	0.82	0.20	0.38	0.16	0.35	0.14				
Severity	0.17	0.24	0.13	0.12	0.20	0.13	0.10	0.10	0.10	0.12			
Subjective Norm	0.66	0.06	0.56	0.63	0.24	0.66	0.20	0.61	0.25	0.44	0.12		
Susceptibility	0.13	0.32	0.08	0.11	0.19	0.11	0.10	0.04	0.10	0.03	0.52	0.18	

Furthermore, discriminant validity was verified by applying the cross-loading criterion (Table 6), showing that an indicator's load on its allocated latent variable is greater than its loads on all other variables.

**Table 6:** Discriminant validity of outer models using the cross-loading criterion.

	ATCov	HBM-Bar	HBM-Ben	Int	PAV-Cal	PAV-Res	PAV-Com	PAV-Conf	PAV-Cons	PBC	HBM-Sev	SN	HBM-Sus
ATCov1	0.95	‒0.21	0.64	0.54	0.16	0.72	‒0.46	0.64	‒0.38	0.40	0.14	0.56	0.15
ATCov2	0.96	‒0.20	0.67	0.54	0.17	0.74	‒0.43	0.66	‒0.39	0.40	0.15	0.58	0.10
ATCov3	0.96	‒0.17	0.67	0.51	0.15	0.69	‒0.40	0.68	‒0.38	0.40	0.14	0.58	0.12
HBM-Bar1	‒0.15	0.75	‒0.08	0.06	0.28	‒0.08	0.28	‒0.21	0.27	0.03	0.20	0.04	0.20
HBM-Bar2	‒0.16	0.84	‒0.10	0.06	0.33	‒0.08	0.32	‒0.28	0.28	0.06	0.15	0.04	0.26
HBM-Bar3	‒0.22	0.81	‒0.18	‒0.11	0.26	‒0.20	0.39	‒0.26	0.36	‒0.10	0.14	‒0.08	0.21
HBM-Bar4	‒0.12	0.83	‒0.08	0.10	0.35	‒0.12	0.25	‒0.30	0.19	0.09	0.15	0.04	0.24
HBM-Bar5	‒0.15	0.81	‒0.13	0.08	0.34	‒0.12	0.21	‒0.32	0.24	0.05	0.18	‒0.03	0.29
HBM-Ben1	0.66	‒0.14	0.92	0.44	0.07	0.64	‒0.35	0.59	‒0.34	0.30	0.09	0.50	0.10
HBM-Ben2	0.53	‒0.15	0.85	0.40	‒0.00	0.57	‒0.23	0.51	‒0.24	0.29	0.08	0.36	0.00
HBM-Ben3	0.65	‒0.12	0.91	0.42	0.10	0.63	‒0.37	0.54	‒0.35	0.28	0.09	0.45	0.08
Int1	0.56	0.03	0.47	1.00	0.21	0.52	‒0.19	0.45	‒0.20	0.82	0.10	0.58	0.11
PAV-Cal1	0.16	0.32	0.10	0.21	0.86	0.20	0.18	0.07	0.11	0.17	0.13	0.15	0.14
PAV-Cal2	0.10	0.35	0.01	0.12	0.85	0.09	0.24	0.04	0.17	0.10	0.13	0.16	0.15
PAV-Cal3	0.17	0.32	0.04	0.19	0.90	0.17	0.25	0.05	0.15	0.20	0.18	0.22	0.17
PAV-Res1	0.69	‒0.16	0.63	0.49	0.16	0.95	‒0.35	0.57	‒0.35	0.33	0.11	0.55	0.09
PAV-Res2	0.75	‒0.15	0.68	0.50	0.19	0.96	‒0.38	0.58	‒0.36	0.35	0.12	0.56	0.11
PAV-Com1	‒0.49	0.42	‒0.43	‒0.23	0.23	‒0.43	0.89	‒0.36	0.61	‒0.18	0.03	‒0.26	0.04
PAV-Com2	‒0.21	0.19	‒0.11	‒0.06	0.16	‒0.15	0.75	‒0.05	0.43	‒0.07	‒0.11	‒0.05	‒0.12
PAV-Com3	‒0.32	0.24	‒0.25	‒0.12	0.23	‒0.27	0.84	‒0.16	0.60	‒0.10	‒0.07	‒0.11	‒0.07
PAV-Conf1	0.62	‒0.37	0.56	0.42	0.02	0.56	‒0.24	0.92	‒0.20	0.30	0.03	0.47	0.01
PAV-Conf2	0.61	‒0.36	0.54	0.41	0.02	0.51	‒0.24	0.92	‒0.18	0.31	0.10	0.48	0.01
PAV-Conf3	0.61	‒0.16	0.54	0.36	0.12	0.53	‒0.25	0.82	‒0.25	0.26	0.09	0.45	0.07
PAV-Cons1	‒0.21	0.12	‒0.18	‒0.09	0.05	‒0.24	0.39	‒0.10	0.71	‒0.07	0.04	‒0.14	0.09
PAV-Cons2	‒0.42	0.36	‒0.40	‒0.22	0.19	‒0.37	0.65	‒0.28	0.90	‒0.15	0.05	‒0.24	0.04
PAV-Cons3	‒0.28	0.29	‒0.20	‒0.14	0.12	‒0.26	0.53	‒0.14	0.80	‒0.08	0.09	‒0.10	0.07
PBC1	0.42	0.01	0.33	0.82	0.19	0.36	‒0.16	0.33	‒0.13	1.00	0.10	0.41	0.03
HBM-Sev1	0.16	0.20	0.07	0.07	0.18	0.14	‒0.05	0.09	0.07	0.07	0.94	0.10	0.43
HBM-Sev2	0.10	0.14	0.12	0.11	0.12	0.06	‒0.01	0.05	0.07	0.11	0.84	0.07	0.37
SN1	0.55	‒0.01	0.48	0.54	0.16	0.55	‒0.20	0.49	‒0.19	0.39	0.08	0.94	0.11
SN2	0.57	‒0.01	0.45	0.55	0.23	0.54	‒0.18	0.50	‒0.21	0.39	0.11	0.94	0.20
HBM-Sus1	0.13	0.30	0.08	0.12	0.19	0.11	‒0.04	0.02	0.06	0.03	0.44	0.16	0.98
HBM-Sus2	0.12	0.28	0.07	0.10	0.15	0.10	‒0.02	0.05	0.08	0.03	0.44	0.17	0.98

**Fig. 3:**
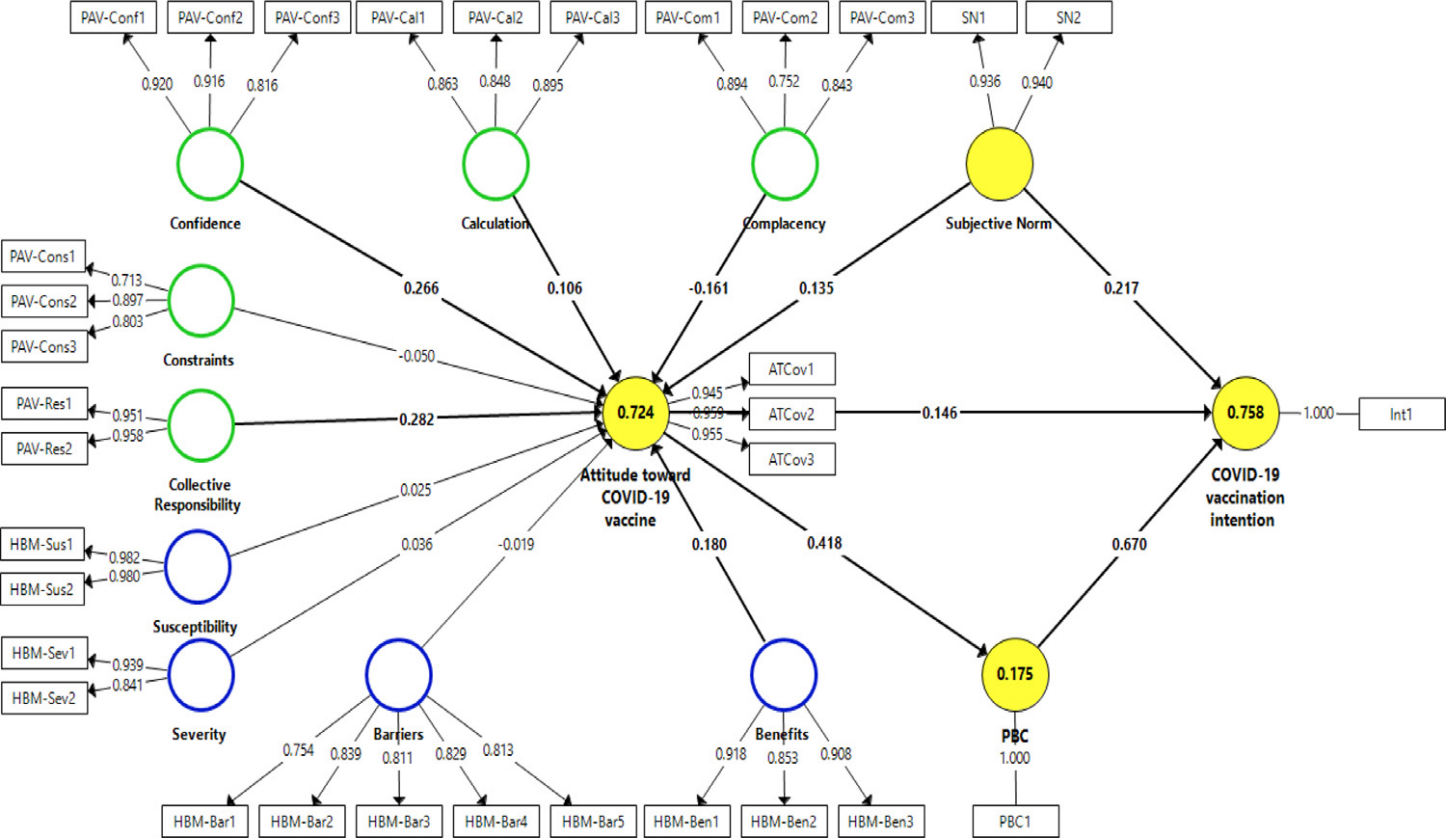
Outer models testing- SmartPLS outputs.

Fig. 3 displays the values of the coefficient of determination (R^2^), indicating that the R-square values of attitude toward COVID-19 vaccination, PBC, and COVID-19 vaccination intention are 0.72, 0.17, and 0.75, respectively.

Table 7 provides the effect of size values of exogenous constructs on endogenous constructs. Further, for all endogenous constructs the Q square value of PBC, attitude toward COVD-19 vaccines, and vaccination intention are higher than zero, being 0.16, 0.64, and 0.73, indicating an acceptable predictive relevance of the model [10] (Table 8). Moreover, the goodness of fit value is 0.67, reflecting a high goodness of fit of the model.

**Table 7:** Evaluation of inner model through effect size criterion.

	Attitude	Intention	PBC
Attitude toward COVID-19 vaccine		0.053	0.212
Barriers	0.001		
Benefits	0.052		
COVID-19 vaccination intention			
Calculation	0.030		
Collective Responsibility	0.113		
Complacency	0.044		
Confidence	0.116		
Constraints	0.005		
PBC		1.453	
Severity	0.004		
Subjective Norm	0.038	0.118	
Susceptibility	0.002

**Table 8:** Q Square values.

	SSO	SSE	Q² (=1-SSE/SSO)
Attitude toward COVID-19 vaccine	969.000	341.296	0.648
Barriers	1615.000	1615.000	
Benefits	969.000	969.000	
COVID-19 vaccination intention	323.000	84.808	0.737
Calculation	969.000	969.000	
Collective Responsibility	646.000	646.000	
Complacency	969.000	969.000	
Confidence	969.000	969.000	
Constraints	969.000	969.000	
PBC	323.000	268.619	0.168
Severity	646.000	646.000	
Subjective Norm	646.000	646.000	
Susceptibility	646.000	646.000	

As detailed in Table 9, the data analysis provides evidence that, from the health belief model, just the perceived benefits (β-value=0.180; p-value= 0.006) have a significant influence on attitude toward COVID-19 vaccination. While the relationships between perceived susceptibility (p-value= 0.493), perceived severity (p-value= 0.290), perceived barriers (p-value= 0.671) and attitude toward COVID-19 vaccines are not significant. In addition, the psychological antecedents of vaccination positively affect individual attitude toward COVID-19 vaccine. More specifically, confidence (β-value = 0.266; p-value= 0.000), complacency (β-value = -0.161; p-value= 0.007), calculation (β-value = 0.106; p-value= 0.009) and collective responsibility (β-value = 0.282; p-value= 0.000) significantly impact on the level of attitude toward vaccination.

**Table 9:** Hypotheses testing- SmartPLS outputs.

	Hypotheses	Original Sample	T Statistics	P Values	Output
**1**	Susceptibility → Attitude toward COVID-19 vaccine	0.025	0.686	0.493	Not supported
**2**	Severity **→** Attitude toward COVID-19 vaccine	0.036	1.060	0.290	Not supported
**3**	Benefits **→** Attitude toward COVID-19 vaccine	0.180	2.784	0.006	Supported
**4**	Barriers **→** Attitude toward COVID-19 vaccine	‒0.019	0.425	0.671	Not supported
**5**	Confidence **→** Attitude toward COVID-19 vaccine	0.266	5.372	0.000	Supported
**6**	Constraints **→** Attitude toward COVID-19 vaccine	‒0.050	0.944	0.346	Not supported
**7**	Complacency **→** Attitude toward COVID-19 vaccine	‒0.161	2.723	0.007	Supported
**8**	Calculation **→** Attitude toward COVID-19 vaccine	0.106	2.636	0.009	Supported
**9**	Collective Responsibility **→** Attitude toward vaccine	0.282	4.313	0.000	Supported
**10**	Subjective Norm **→** Attitude toward COVID-19 vaccine	0.135	2.514	0.012	Supported
**11**	Attitude toward vaccine **→** PBC	0.418	7.757	0.000	Supported
**12**	Attitude toward vaccine **→** Vaccination intention	0.146	4.029	0.000	Supported
**13**	Subjective Norm **→** COVID-19 vaccination intention	0.217	5.522	0.000	Supported
**14**	PBC **→** COVID-19 vaccination intention	0.670	14.759	0.000	Supported

**Fig 4:**
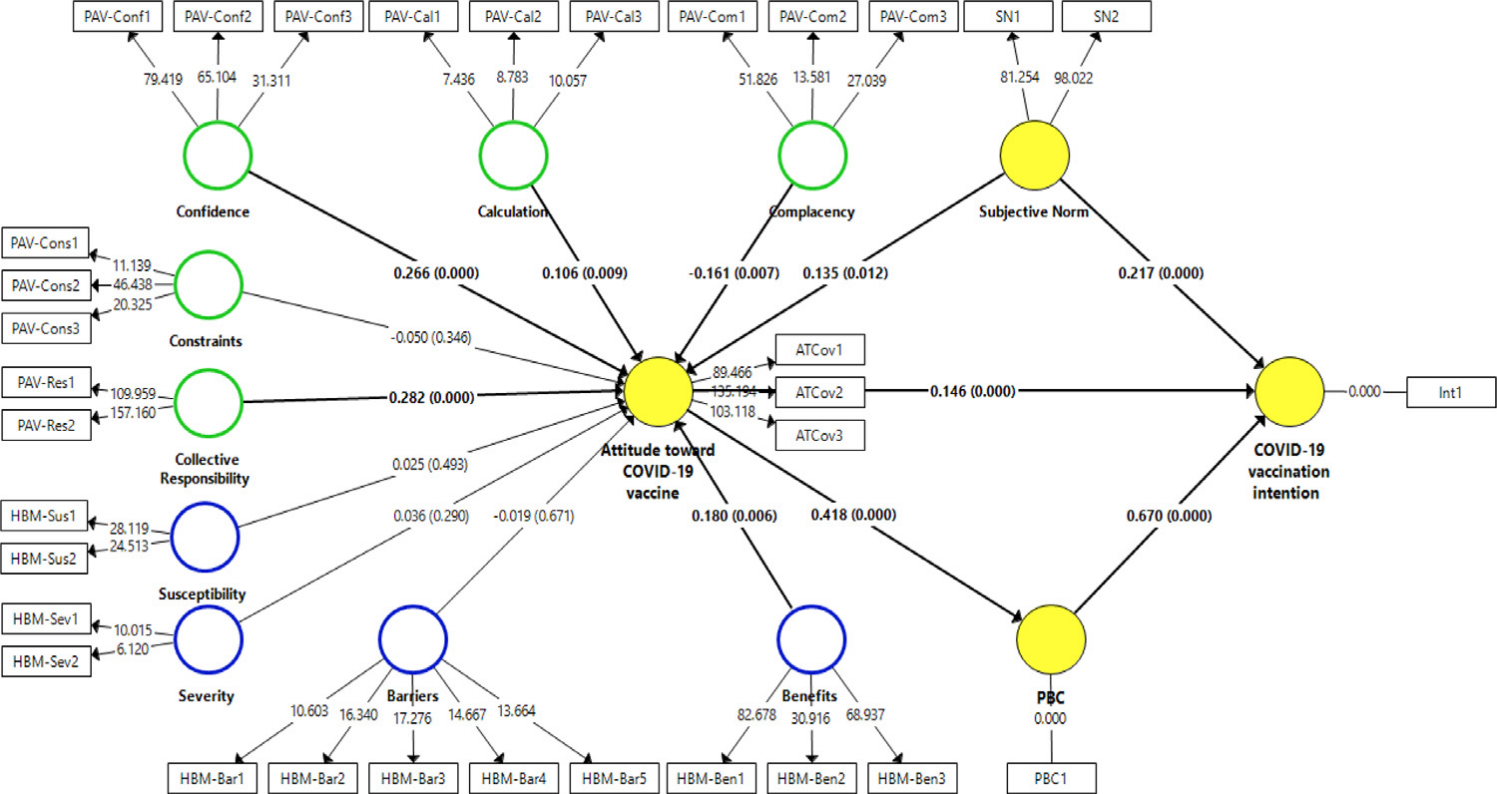
Hypotheses testing- SmartPLS outputs.

Data analysis using the PLS approach confirms the validity of the TPB, indicating that subjective norms have a positive impact on individuals' attitude (β-value = 0.135; p-value= 0.012) and intention regarding COVID-19 vaccination (β-value = 0.217; p-value= 0.000). Furthermore, attitude toward vaccination positively and directly affect on the perceived behavioural control (β-value = 0.418; p-value= 0.000) and the individual vaccination intention (β-value = 0.146; p-value= 0.000). Lastly, a high degree of perceived behavioural control (β-value = 0.670; p-value= 0.000) help to enhance individual intention to receive COVID-19 vaccination (Fig. 4).

## Ethics Statement

This survey data passed the ethical review by Bioethics Consultative Commission Faculty of Sciences Agadir, Ibn Zohr University, Morocco. (No: ER-BS-03/2022-0001). The survey data was conducted according to the Declaration of Helsinki.

## CRediT Author Statement

**Mohammed Abdessadek:** Writing - Original draft preparation, Investigation, Data curation; **Omar Boubker:** Conceptualization, Methodology, Software, Data Analysis; **Hayat Ben-Saghroune, Imane Iken, Houssni ABID, Belkassem El Amraoui, Youssef Khabbal:** Project Administration, Reviewing and Editing.

## Funding Resources

This study received no specific grant from any funding agency in the public, commercial, or not-for-profit sectors.

## Declaration of Competing Interest

The authors declare that they have no known competing financial interests or personal relationships, which have or could be perceived to have influenced the work reported in this article.

## Data Availability

Factors affecting COVID-19 vaccination intention (Original data) (Mendeley Data).
